# Early detection of dust accumulation on solar energy modules using computer vision and machine learning techniques

**DOI:** 10.1038/s41598-026-37020-0

**Published:** 2026-02-12

**Authors:** Sara Hesham, Mohamed Elgohary, Mariam Massoud, Nouran Adel, Omar Elmahy, Sameh Abdellatif

**Affiliations:** The Electrical Engineering Department and FabLab, at the Centre for Emerging Learning Technologies, CELT, British University in Egypt (BUE), Misr-Ismalia Desert Road, PO Box 43, El-Sherouk City, Cairo 11837 Egypt

**Keywords:** Dust accumulation, Solar energy, Machine learning, Computer vision, Dynamic cleaning, Energy science and technology, Engineering, Mathematics and computing

## Abstract

This paper presents an innovative AI-driven solution for the early detection of dust accumulation on solar energy modules, leveraging computer vision and machine learning techniques. The study identifies two significant gaps in existing literature: the impact of dataset quality on research outcomes and the predominance of binary classification models, which limit the analysis of dust levels on photovoltaic (PV) modules. To address these gaps, we propose a sophisticated system that utilizes a visual dataset of continuously monitored images captured by a Raspberry Pi camera, alongside a raw dataset from the inverter tracking real-time energy production metrics. Our model is trained using machine learning algorithms to optimize cleaning patterns dynamically, maximizing energy output while minimizing operational costs. The results indicate that the AI-powered system enhances PV performance and contributes to cost reductions, ultimately promoting sustainability in solar energy production. The cleaning classifier automates condition-based cleaning, reducing the need for routine inspections and preventing up to 30% energy loss due to dust accumulation. The system achieves a cleaning efficiency of 1.23, reflecting a 23% increase in energy production compared to traditional periodic cleaning methods, resulting in a cost saving of $2,023. Furthermore, the implementation of the WattsUp mobile application facilitates user interaction by allowing monitoring of solar panel status and maintenance instructions, enhancing user engagement with the system. The application design emphasizes user trust and participation, featuring onboarding elements that highlight the importance of solar energy management in combating climate change. This comprehensive approach not only demonstrates the feasibility of a dynamic cleaning model but also establishes a payback period of less than a year, underscoring the economic viability of the proposed system in real-world applications.

**Supplementary Information:**

The online version contains supplementary material available at 10.1038/s41598-026-37020-0.

## Introduction

Solar energy is a key renewable source, due to its abundant availability and minimal environmental impact^1^. Solar power has become one of the most promising alternatives to fossil fuels^2–4^. Electricity from photovoltaic (PV) panels has revolutionized energy production by converting sunlight directly into electricity, thereby supporting both remote and urban energy infrastructures^2^. The dirt collected on solar panels causes a reduction in power output as sunlight is blocked^5^. This phenomenon, often referred to as soiling, can significantly lower the efficiency of PV systems when contaminants accumulate on the surface^3^. As efficiency drops, maintenance teams are compelled to perform frequent cleaning and repairs, which further drives up operational expenses^2^. Most of the dust issue was seen in dry areas where frequent cleaning is needed to maintain them. Arid regions, characterized by high dust concentrations and low precipitation, tend to experience accelerated soiling, necessitating more rigorous maintenance protocols. Arid regions, characterized by high dust concentrations and low precipitation, tend to experience accelerated soiling, necessitating more rigorous maintenance protocols^2^. While reviewing the literature, various cleaning methods were introduced, including dry cleaning, wet cleaning, and detergent cleaning^4^. Moreover, it was reported that environmental changes—such as fluctuations in temperature, humidity, and wind speed—play a significant role in both the rate of dust accumulation and the effectiveness of each cleaning method. For instance, high humidity can cause water droplets to bond dust to the panel surface, making dry cleaning less effective, while in windy conditions, dry cleaning may suffice by dislodging loosely attached particles without the need for water^5^. Accordingly, a dynamic cleaning schedule with various cleaning alternatives may be seen as the optimum way for maximizing the system energy yield and minimizing the cost.

Referring to the literature, researchers have applied advanced techniques, including but not limited to computer vision, machine learning, and deep learning, to effectively track the dust accumulation with correlated effect on energy production and cost^2^. The use of machine learning (ML) for predictive maintenance of PV systems has opened the door to the automated identification and prediction of performance losses associated with soiling^6,7^. ML algorithms are surpassing conventional statistical or rule-based process monitoring systems by learning rich patterns in data of diverse origin, such as time-series electrical outputs, environmental sensors, or images^8^. As an example, hybrid approaches that integrate Long Short-Term Memory (LSTM) networks with K-Nearest Neighbors (KNN), such as the hybrid LSTM-KNN algorithm, have shown remarkable accuracy of ~ 98.22% in predicting energy losses due to dust, exceeding the accuracy of LSTM (95.51%) and KNN (61.49%) when used in isolation^9^. The ability of ML to closely model panel output for soiled conditions was demonstrated in Badli, India, where the R^2^ and mean square error (MSE) values for the six months of real-time soiling ratio data using stacked and bidirectional LSTM models were 0.991 and 0.0078^10^. These approaches use certain environmental parameters, including sunshine hours, humidity, temperature, and solar radiation, with solar radiation having been confirmed as the greatest influencing parameter of soiling-induced losses^11^. Other important techniques are ensemble methods and feature-based classifiers. Random Forests^12,13^, support vector machines (SVMs)^14^, and convolutional neural networks (CNNs)^15^ have been trained on structured performance data and features from images, achieving an F1-score of approximately 0.93 in soiling classification^16^. Of note, the use of data augmentation, including synthetic sample generation and transformations of time-series data, has provided considerable improvement in model generalization, particularly within ensemble models, where performance on the clean/dirty classification task saw noticeable improvements when using augmented inputs^16–18^.

Alternatively, automating dust detection on PV modules via computer vision has transitioned from conventional texture analysis techniques to advanced deep learning architectures capable of achieving accuracy, scalability, and robustness across varying conditions^19,20^. Initial methods such as the gray-level co-occurrence matrix GLCM + local binary pattern (LBP) combined with a support vector machine obtained an accuracy of about 94.3% in desert-like environments, and despite a challenging and variable lighting, this showed that proper feature descriptions may still be useful in environments with limited resources^21^. But most of these systems adopt CNN architectures, and a model specifically designed for this purpose, SolNet, obtains 98.2% accuracy on a custom dataset of clean against dusty panels^21^. Other pretrained networks, such as DenseNet169, have also been successfully used: when used as a feature extractor and combined with linear SVM, it achieved 86.8% accuracy, showing the capabilities of learned features combined with simple classifiers^22^.

Such novel approaches deploy a vision-based UAV system at a large scale. When tested with the 3D-augmented synthetic data, results from the improved YOLOv8 models (YOLOv8-DSDA) exhibited significantly higher mAP and recall performance metrics, as they successfully mitigated common UAV factors such as differing angles and lighting^23^. Also, multimodal CV systems fuse infrared thermography with visible light to identify hotspots, shading, bird droppings, and other defects, and have achieved over 98% AP@0.5 for module detection and ~ 70% mAP for visible-spectrum anomalies using models such as YOLOv3^24^. Novelties include attention-based Vision Transformers (ViT,s), which are reported to reach > 97% accuracy in the identification of surface defects related to dust, cracks, delamination, and bird droppings, illustrating some further versatility in application to both PV and wind^24^.

Following the scanned literature above, two main literature gaps can be observed. The two observed gaps in the literature related to this research topic are: the dataset used to conduct research impacts results, whether artificial or natural. Moreover, most models in the available research focus on binary classification, which has its limitations. This highlights a clear gap in the literature regarding the quantitative analysis of dust multi-level classification of dusty PV modules into different dust levels. Alternatively, a continuous regression model could provide a percentage indicator of dust accumulation, the latter two allowing for a direct correlation with the module’s power output. In alignment with the identified gaps in the existing literature, we propose a sophisticated, AI-driven solution designed to optimize dust tracking mechanisms and establish a dynamic cleaning model for photovoltaic systems. This innovative approach leverages two distinct datasets: a visual dataset comprising continuously monitored images captured by a Raspberry Pi camera, which assesses the dust accumulation on PV cells with the aid of a computer vision model, and a raw dataset extracted from the inverter that tracks real-time energy production metrics. By employing machine learning (ML) algorithms, our model is trained on real data to dynamically determine the most efficient cleaning patterns, thereby maximizing energy output while minimizing operational costs. This intelligent system not only enhances the efficiency of PV performance but also contributes to significant cost reductions, ultimately leading to improved sustainability in solar energy production.

The AI-powered system introduced in this work represents not merely an operational enhancement but an essential advancement in solar energy management. The cleaning classifier is an innovative solution designed to address sustainability and operational challenges within this field. Traditional scheduled inspections or routine cleanings often prove inefficient and time-consuming. In contrast, the classifier facilitates condition-based cleaning by automating critical decision-making processes, thereby eliminating the need for continuous human oversight. Moreover, dust accumulation can significantly impair the efficiency of solar panels, resulting in reductions of up to thirty percent in performance. The classification system enables early detection of such degradation, ensuring that panels are cleaned only when a decline in performance is identified, thus preventing unnecessary energy loss. Additionally, excessive cleaning incurs avoidable costs related to water usage and labor. Conversely, infrequent cleaning leads to lost energy production. The classifier optimizes the cleaning process by identifying the ideal balance between maintenance efforts and operational profitability. Manual inspections on a large scale within solar farms are impractical. AI classification allows for the simultaneous monitoring of hundreds or even thousands of panels without added complexity, ensuring system-wide reliability. This paper advocates for intelligent, efficient, and sustainable maintenance of solar energy systems through the incorporation of the classifier into the system architecture. This approach aligns with the overarching objectives of renewable energy optimization while simultaneously enhancing the sustainability of solar infrastructure.

## Hardware setup

**Fig. 1:**
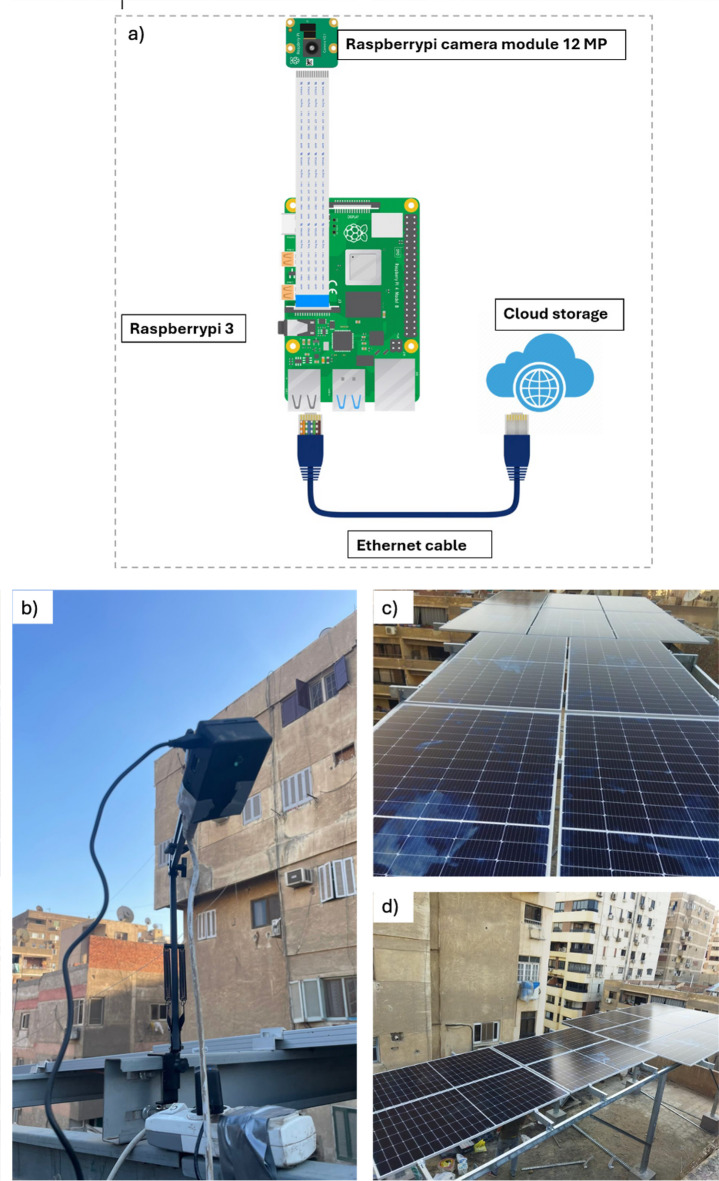
(**a**) Raspberry Pi camera terminal node schematic, (**b**) real node with 3D printed cover, (**c**) and (**d**) samples of positions used to collect images.

**Fig. 2:**
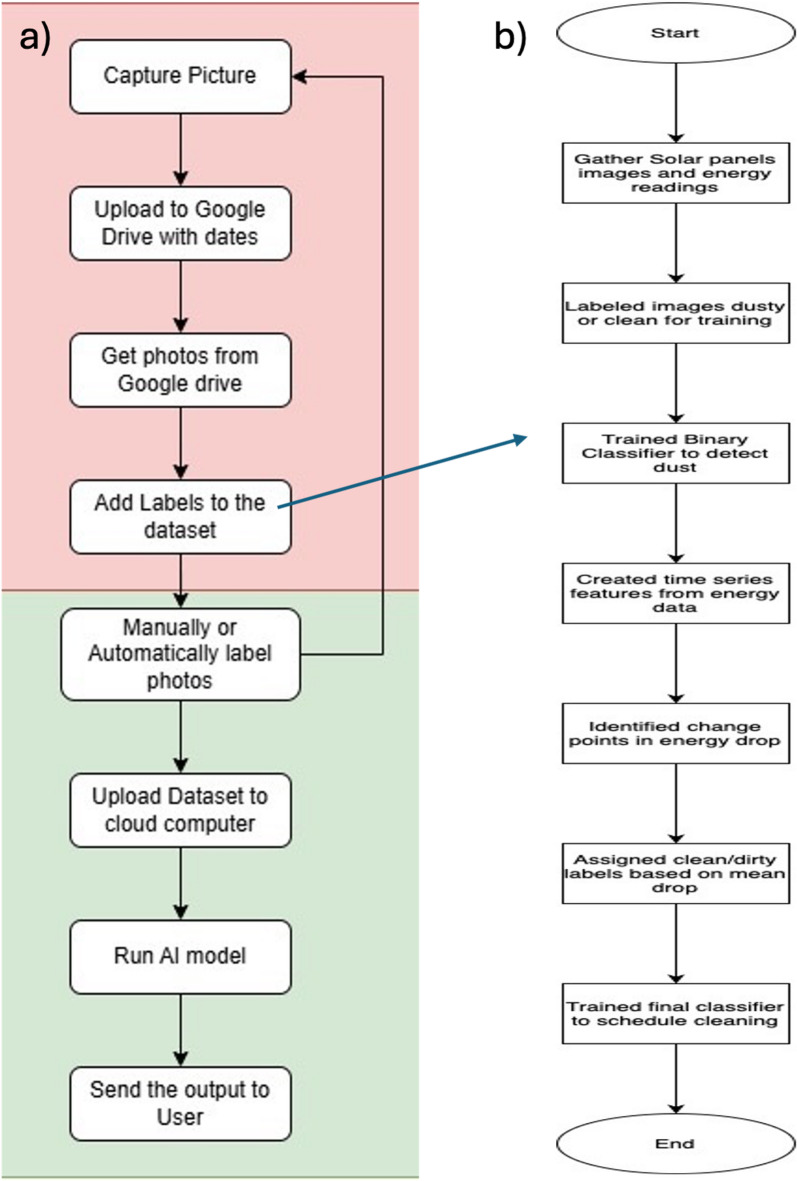
(**a**) Workflow for solar panel cleaning detection, and (**b**) pseudo-labeling methodology pipeline.

Since the proposed approach is relatively new and to overcome the lack of publicly available datasets relating to solar panel energy production and visual imagery, a 5-kW photovoltaic system was installed specifically to facilitate experimental data collection and small-scale prototype implementation in Cairo, Egypt. See our implemented systems in^25,26^. Such a homebrew arrangement allows obtaining synchronized energy readings and images in a real-world scenario. The primary data collection started on November 27, 2023, and ended on May 22, 2025, during which 536 days were utilized. Data is continuously being collected to keep growing and enlarging the dataset to keep experimenting and refining the model. The creation of a hardware prototype was carried out with the main goal of capturing and storing photographic data to be analyzed later. The system consists of a 12-megapixel Raspberry Pi Camera Module, Raspberry Pi 3 Model B, a power supply unit, and an Ethernet cable to maintain a steady network connection. The hardware nodes were placed on a tripod stand and put into a 3D-printed case with a special design that is resistant to water and dust and allows the device to be used outdoors all the time, cf. Figure 1. The images are automatically uploaded to Google Drive cloud storage to enable safe storage and remote access of the images to continue with the processing. The development of multiple hardware prototypes aimed at training a generalizable computer model and possessing a varied dataset was carried out. These systems were set with the cameras at various distances from the solar panel. Each prototype collected 200 images daily at different times to consider the effect of lighting and shadows at different times of the day. Such a multi-view, high-frame imaging system was also aimed at increasing the robustness of the model in a variety of visual circumstances. The entire process is flowcharted in Fig. 2a.

## Dataset collection, cleaning, and preprocessing

**Fig. 3:**
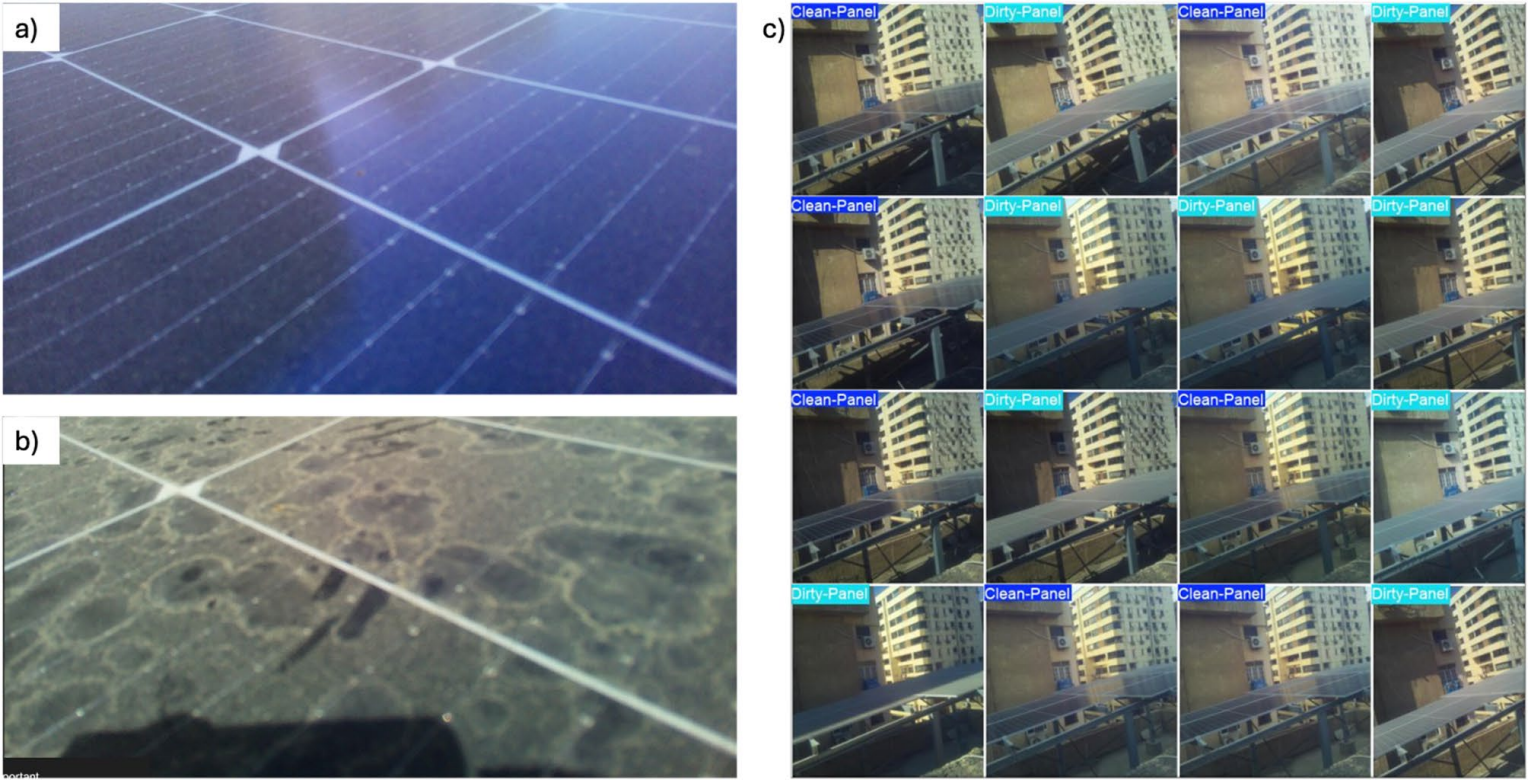
(**a**) Clean PV image, (**b**) Dusty PV image, and (**c**) labeling process.

In the current study, two main datasets were gathered. Firstly, a visual dataset of clean and dusty panels. Secondly, a dataset exploring the daily energy yield output from the inverter, see the inverter dataset in the Supplementary Material. The dataset of dusty and clean solar panels was custom-made by supplementing 17,000 labeled images taken with the developed hardware prototype, as described in the previous section, Section “Hardware setup”, see Fig. 3. The entire dataset is available through the data availability section using an online repository. The data neutrality was considered to avoid any bias. The hybrid strategy, for integrating images with different positions, was pursued to bring diversity in the dataset as well as to achieve better generalization of the model on the different environmental conditions and types of panels. This is because the images of the prototypes are included, and therefore, the model is presented to the actual solar panels that are used in the deployment, and hence, the accuracy of the predictions should be enhanced in the real world. Since the rate at which the images were captured was very high and the process of dust accumulation was slow, many images collected were visually redundant. To maintain dataset quality and prevent unwarranted duplication, the image hashing method was used to automatically detect and delete near-duplicate images. This preprocessing procedure led to a cleaned-up version of unique images, which were further subjected to manual annotation. The last group of unique images amounts to 17,000 images. The dataset was loaded to Roboflow to be labelled. The annotated images in the form of the sample in the figure comprised 10,300 images in which the solar panel looked dusty, and 6700 in which it looked clean. Class imbalance was obvious, as the dusty images of solar panels were nearly twice as many as the clean ones.

All the captured images are preprocessed with a standard pipeline to make the models consistent and perform better. Automatic orientation correction and uniform resizing to a 640 × 640 pixel resolution were applied. The auto-orientation becomes important in ensuring geometric consistency throughout the dataset, as is present in the real-world deployment scenario, where the Raspberry Pi cameras are likely to take photos at different angles depending on the environmental conditions or by manual operation. Rotated or misaligned images may harm the performance of a convolutional neural network (CNN) to identify relevant spatial features, i.e., surface texture variations or local dust patterns. The automatic orientation correction aligns all the images to a canonical viewpoint, improving the capacity of the model to learn spatially coherent representations, see Fig. 3.

Resizing to 640 × 640 pixels serve several purposes. First, it normalizes the dimensions of inputs to the deep learning model, which is crucial in the case of batch processing and fixed input demands of most CNN architectures. Second, this resolution balances between the preservation of important visual details, e.g., fine-grained dust settlement or surface reflections, and computational efficiency. There can be a higher-resolution image, which will have more information but will occupy more memory and take time to train, and an image that is excessively downscaled can lose its salient features, which can be used to classify the image correctly. The resolution that was selected allows for the preservation of the visual cues that are related to the perception of the differences between the dusty and clean states of solar panels, without the high computational cost. Collectively, these preprocessing procedures make the learning pipeline more robust and reliable by making the data uniform and keeping discriminative visual information. This is especially relevant in solar panel monitoring systems where the visual contrast between dusty and clean conditions can be small and dependent on perspective, lighting, and resolution.

## Dataset processing and training

**Fig. 4:**
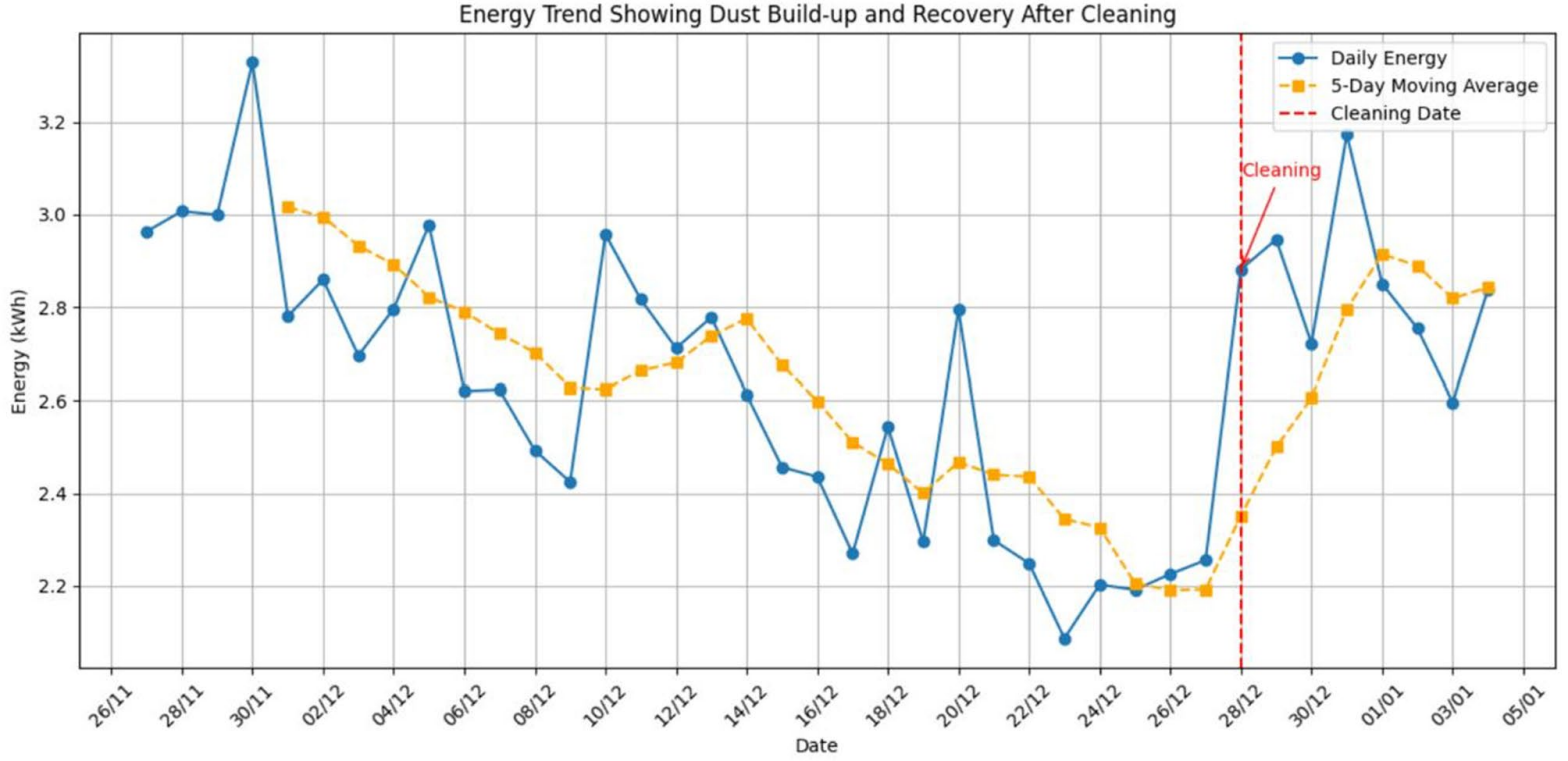
Energy yield from the PV system daily.

To train our computer vision model, we divided the image dataset, using 70% of the images for training, 15% for validation, and the final 15% for testing. This method ensures the model learns the patterns correctly before we test its performance on a separate set of images it hasn’t seen before. Alternatively, the energy output was measured continuously through the system DC-AC inverter, and the daily energy values were used in the analysis, as they better show the slow effects of dust build-up than the minute- or hour-scale data. Figure 4 shows a sample of daily energy data, which clearly shows a definite decreasing trend in the energy generation and then a sudden rise that can be attributed to a known cleaning event. This cyclical behavior of a drop in readings followed by an increase after cleaning was both distinct and present throughout the data. It is one of the main indicators of the impact of soiling on panel performance. The classifier was developed taking advantage of this trend, and the data was annotated accordingly, as it will be explained later in this chapter. The cleaning classifier aims at deciding on a given day whether cleaning is necessary, given the input features, which consist of energy output and the resulting dust state.

The cleaning classifier depicts a conceptual schematic for the primary decision algorithm of the proposed dust detection pipeline: the AI-based cleaning classifier. The classifier operates as a binary classifier that takes input and, based on real-time conditions, ascertains whether cleaning is needed for the solar panel. The input consists of two modalities: image data, captured by the Pi camera, which represents the visual state of the panel surface and is sent to the computer vision model. Secondly, the energy data: the data are collected daily from the photovoltaic system and represent the performance of the panel, which serves as the basis to identify declines that may be due to dust on the panels. The combination of these inputs is fed into an artificial intelligence model, which has been trained to identify patterns that relate to soiling. The final model selection for the image classification model was a Vision Transformer (ViT) architecture, which achieved the best results for accuracy and is effective at identifying subtle visual differences. Energy classification was done in parallel using classical algorithms, mainly Random Forest and Logistic Regression, based on pseudo-labeled data that identified energy trends because of energy change point detection. This is described in detail in the coming subsections.

### Feature engineering and data segmentation

Besides visual data analysis, another novelty of this paper is the model applied, which targets non-visual indicators of a time series of daily energy production of photovoltaic (PV) modules. This is an improved way of detecting dust through analysis of the performance trends when visual data may not give the best answers. We extract a few derived features out of the raw time-series data to illustrate the patterns behind it: Moving Average: A trailing moving average was taken on a 3-day rolling window. This can reduce short-term variability and bring longer-term trends in energy production, which is standard practice in time series analysis and prediction. First Derivative (Daily Change): By examining the daily changes, the model can identify sudden decreases in energy generation rate that can be related to dust accumulation events. Lagged Values: Lagged features were added to the energy outputs to represent the temporal dependencies and memory effects in the performance of the panels (i.e., outputs of past days).

With the Python library Ruptures, we divided the time series into segments that may be understood as indicating the constant performance, the possible dust accumulation, or the cleaning events. This tool determines structural break points in time when the statistical characteristics of the data change significantly. Using the PELT (Pruned Exact Linear Time) algorithm, we may divide the series into successive regimes, each of which is probably related to a period of dust deposition, a cleaning episode, or simply noise variations. We compute the daily change average in every segment, denoted by Ruptures. When this average is negative, then that indicates a decline in output, and we call that section Potential Cleaning, which means it is probably dirty. Conversely, when the average is neutral or positive, we refer to it as, No Cleaning This smart pseudo-labeling trick enables us to generate training labels automatically without manual intervention, which increases the scalability, and we can develop supervised models with minimal human intervention. This combination of classic feature engineering, change-point detection, and pseudo-labeling allows addressing both gradual and abrupt performance changes effectively. It enhances the precision of dust identification and coincides with the plans in the PV literature that emphasize the benefits of change-point strategies to identify soiling and optimize cleaning intervals^7^.

### Pseudo-labelling logic

Having segmented the time series data using the Ruptures algorithm, we can assess each segment to determine whether it warrants a cleaning label. The logic we employ is as follows: when the average energy decrease in a segment is negative, it indicates that the panel’s performance is monotonically declining, likely due to dust accumulation. In such cases, we label the segment as “Potential Cleaning.” Conversely, if the mean change is positive or near zero, we infer that the performance is stable or even improving, thus assigning the label “No Cleaning.” This methodology exemplifies pseudo-labeling cf. Figure 2b, where the system generates training labels based solely on the inherent data structure, eliminating the need for manual annotation. This approach has proven particularly effective in monitoring solar panel performance, as obtaining precise labels—such as the last cleaning date—can be challenging in real-world scenarios. Our application of pseudo-labeling combines performance decrements with a domain-specific model of soiling patterns, enhancing the accuracy of our cleaning predictions.

To illustrate this concept, consider the following example, for segment #1: average energy output decreases from $E$ Wh to ${E}{\prime}$ Wh over a successive number of days. The average energy change is ${E-E}{\prime}$ Wh, indicating a decline in performance by Δ. If this Δ is greater than a specific threshold, the segment is labeled as Potential Cleaning.” In segment #2, the average energy output remains stable at $E$ Wh, with fluctuations between ± ∂ Wh. Herein, the average energy change is minor, suggesting stable performance. This segment is labeled as “No Cleaning.” By implementing this pseudo-labeling logic, we can effectively track the performance of solar panels without the burden of manual data collection, thereby enhancing the efficiency of our cleaning model. This approach not only streamlines the process but also contributes to improved model generalization, particularly in scenarios where labeled data is scarce. Visual examples are attached in the Supplementary Material.

### Algorithms used

Upon segment labelling, we proceed to train two ML models using the pseudo-labeled dataset: Random Forest (RF) and Logistic Regression (LR). We adopted Random Forest (RF) as it performs exceptionally well on non-linear relationships and is robust against overfitting, particularly in cases of structured data like engineered time-series features. It is frequently used in renewable energy predictive tasks, RF^12,13^. Alternatively, Logistic Regression serves as our baseline classifier owing to its straightforwardness, high interpretability, and efficiency in binary classification tasks. The models were trained on the features derived during the feature engineering stage, such as moving averages, lagged values, and first-order differences^27^.

### Models evaluation

The validation of ML models is based on a set of evaluation metrics that measure the efficiency and effectiveness^28^. The most common evaluation metrics are described here. Initially, accuracy is treated as one of the most evaluating parameters for ML models. Accuracy is defined as the overall correctness, the percentage of write predictions against all the predictions. It can be described as^28^:

1 $$Accuracy= \frac{TP+TN}{TP+FP+TN+FN}$$

where True Positives ($TP$): The model says true while the real instance is true, True Negative ($TN$): The algorithm says false while the real instance is false, False Positives ($FP$): says true while the real instance is false, considered as Type 1 Error, and False Negatives ($FN$): The model says false while the real instance is true, considered as Type 2 Error. Another important evaluating parameter is the precision, defined as the true positives out of predicted positives; it indicates how many correct true predictions are made in all true detections, and is given by^28^:

2 $$Precision= \frac{TP}{TP+FP}$$

Moreover, the recall as an evaluating factor is determined as the true positives out of actual positives; it indicates how many correct true instances are among all true instances, using the equation^28^:

3 $$Recall= \frac{TP}{TP+FN}$$

The harmonic mean of precision and recall; presents the mean value of the model’s precision and recall, which is known as the F1-Score, and formulated by^28^:

4 $$F1= \frac{2 \times Precision\times Recall}{Recall+ Precision}$$

Under the same context, the AUC-ROC refers to the area beneath the receiver operating characteristic curve, which summarizes a model’s ability to distinguish between classes. Finally, we utilize the confusion matrix, which is a table used to evaluate how well an algorithm performs by showing the counts of correct and incorrect predictions, broken down into categories^28^.

### Economic considerations

Although applying the evaluation matrix outlined in Section "Models Evaluation" is essential for assessing the performance of the machine learning models utilized in this work, a key consideration in this study is the economic impact of the system. Specifically, the current study introduces a dynamic cleaning model that aims to enhance the techno-economic behavior of a photovoltaic system compared to the traditional periodic cleaning schedule. To comprehensively evaluate the benefits of the proposed dynamic cleaning model, it is imperative to compare the system’s performance and the operational cleaning costs associated with the proposed model against those of the conventional bare system. This comparison incorporates both the technical aspects related to the recorded enhancements in energy yield and the economic implications of the associated costs for the proposed system, concerning the increased revenue generated.

Technically, the annual enhancement in the energy yield before and after applying the proposed smart cleaning scheduling model can be determined as:

5 $${\eta }_{cleaning}= \frac{\sum_{i=1}^{365}{E}_{d,i}- \sum_{i=1}^{365}{E}_{P,i}}{\sum_{i=1}^{365}{E}_{P,i}}$$

where ${\eta }_{cleaning}$ is an indicator to assess the dynamic cleaning model performance; a positive number indicates enhancement in the annual energy production, zero refers to no change, while negative values indicate energy reduction. The daily energy yield in kWh after applying the dynamic model, and for the bare periodic cleaning model, are noted as ${E}_{d,i}$, and ${E}_{p,i}$ respectively, with $i$ referring to the day count. As highlighted earlier, the technical side is not the only reference for system assessment. To ensure the economic feasibility of the system, the overall annual savings due to system implementation is formulated as:

6 $${C}_{saving}= {N}_{p} \times { OC}_{cleaning }+\left(\sum_{i=1}^{365}{E}_{d,i}- \sum_{i=1}^{365}{E}_{P,i}\right){ \times C}_{Tariff}- {N}_{node} \times {Cap}_{node}- {N}_{d} \times { OC}_{cleaning }-{ OC}_{cloud}$$

where ${C}_{saving}$ represents the total annual savings after applying our proposed algorithm, ${N}_{p}$ is the total number of cleaning processes across the entire year, ${OC}_{cleaning}$ is the operational cost required for a single cleaning process, including the labor cost and other indirect costs, such as water consumption. Alternatively, ${C}_{Tariff}$ indicates the electricity tariff per kWh, ${N}_{node}$ is the number of hardware prototyped nodes, and ${Cap}_{node}$ is the capital cost per node. On the other hand, the number of cleaning processes through a year after applying the proposed model is indicated as ${N}_{d}$, and ${OC}_{cloud}$ summaries the cloud storage and computing cost. The economic analysis not only emphasizes the importance of evaluating the ML model’s performance through the evaluation matrix but also underscores the necessity of understanding the economic implications of implementing a dynamic cleaning model. This analysis provides a comprehensive view of how the proposed system enhances both the energy yield and the overall economic viability of PV systems, ultimately contributing to more sustainable solar energy management practices.

## Results and discussions

This section presents the empirical results of the computer vision and ML models developed for dust detection. It begins by detailing the performance of the evaluated models, YOLOv11n-cls, YOLOv11x, and ResNet, supported by quantitative metrics and visual evidence. Following the presentation of results, a thorough discussion analyzes these findings, comparing the models’ effectiveness and exploring the implications of the dataset strategy on their performance. The section concludes by contextualizing these results within the proposed model’s broader goal of creating an intelligent, automated solar panel maintenance system.

### Computer vision models performance results

The cornerstone of the visual detection system is a model’s ability to accurately differentiate between a clean and a dusty solar panel. To achieve this, a carefully curated dataset was developed. As outlined in Sections “Dataset collection, cleaning, and preprocessing”, and “Dataset processing and training”, the training data were strategically collected to represent the two most critical states: images of the panels on the day they were professionally cleaned (100% clean) and images from the day immediately preceding the cleaning (100% dirty). This approach, totaling approximately 17,000 images, provided the models with an unambiguous signal for what constitutes a panel in need of maintenance. The models were trained on this dataset, and their performance was evaluated based on their ability to classify unseen images from the validation set. The primary metric used for evaluation was accuracy, which provides a comprehensive view of classification performance. The data recorded highlights the performance accuracy of three different models in a comparative analysis. The ResNet model achieved an accuracy of 87.0%, indicating its effectiveness in the given task. Meanwhile, the YOLOv11n-cls model slightly outperformed ResNet with an accuracy of 88.0%, showcasing its enhanced capability for classification tasks. The YOLOv11x model demonstrated the highest accuracy among the three, reaching an impressive 90.7%. This progression in accuracy suggests that the YOLOv11x model is particularly well-suited for the target application, outperforming both ResNet and YOLOv11n-cls, and emphasizes its potential for superior performance in future implementations. In this study, segmentation networks such as U-Net and DeepLab were not considered primarily due to the specific nature of the task, which focuses on determining whether solar panels are clean or dusty, rather than requiring precise pixel-level delineation of dust on the panel surface. While segmentation models excel at understanding the contextual and spatial relationships within images by classifying individual pixels, the goal here was to achieve efficient classification between two distinct states—clean and dirty—rather than segmenting and labeling dust particles.

The results indicate that the computer vision approach is highly effective for detecting dust on solar panels, with the YOLOv11x model achieving a notable accuracy of 90.7%. This level of performance provides a robust foundation for the entire automated cleaning system, ensuring that maintenance decisions are based on reliable, data-driven insights. The success can be attributed to both the choice of model architecture and the strategic construction of the dataset. A comparative analysis of the models reveals important trade-offs. The YOLOv11x model, being the largest and most complex of the three, unsurprisingly delivered the highest accuracy. Its architecture is adept at learning intricate features and spatial relationships, which allows it to effectively discern the subtle textures and color shifts associated with dust accumulation. However, the YOLOv11n-cls (“nano”) model was remarkably competitive, achieving an accuracy of 88%. The significance of this finding lies in the balance between performance and computational cost. The nano model is substantially smaller and faster, making it a more practical choice for deployment on resource-constrained edge devices like the Raspberry Pi used in the prototype. For a final implementation, the 2.7% accuracy gain from the x-large model must be weighed against its higher computational demands. Both YOLO models outperformed the general-purpose ResNet classifier (87% accuracy). This suggests that architectures designed with object detection principles in mind are inherently better suited for this specific task. YOLO models are trained to identify where an object is and what it is, forcing them to focus on the panel’s surface features rather than the entire scene. ResNet, by contrast, processes the whole image, and its performance may have been slightly diluted by irrelevant background elements like the sky, surrounding structures, or shifting shadows, even with a tightly cropped dataset. Regarding the comparison of the YOLOv11x model—despite its relative heaviness—with the lightweight ResNet and YOLOv11n models, this choice highlights the performance trade-offs inherent in different model architectures. The YOLOv11x model, while computationally intensive, exhibited superior accuracy due to its ability to learn intricate features and spatial relationships, making it particularly well-suited for detecting nuanced signs of dust accumulation.

**Fig. 5:**
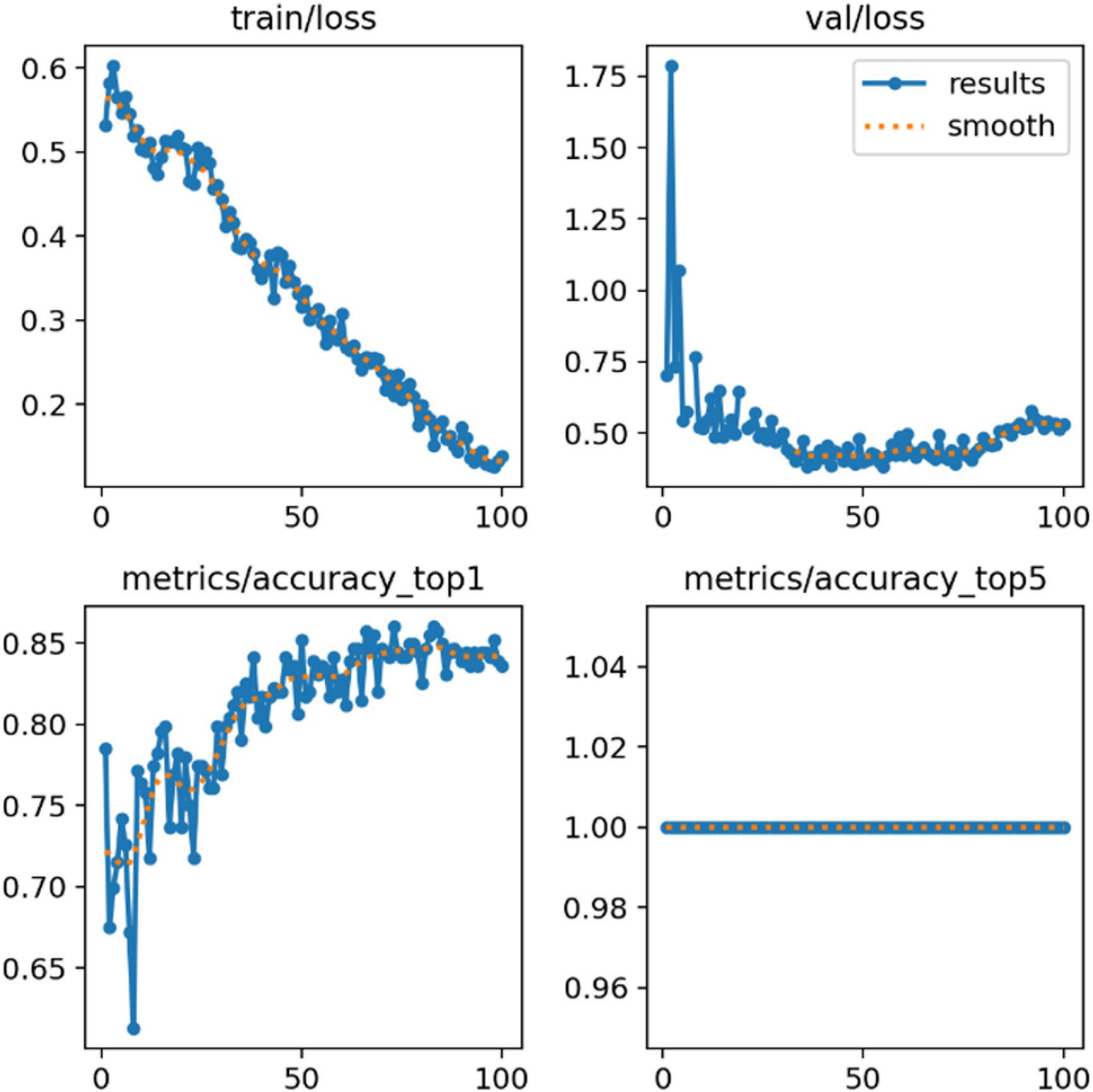
Training and validation accuracy/loss curves for the YOLOv11x mode.

**Fig. 6:**
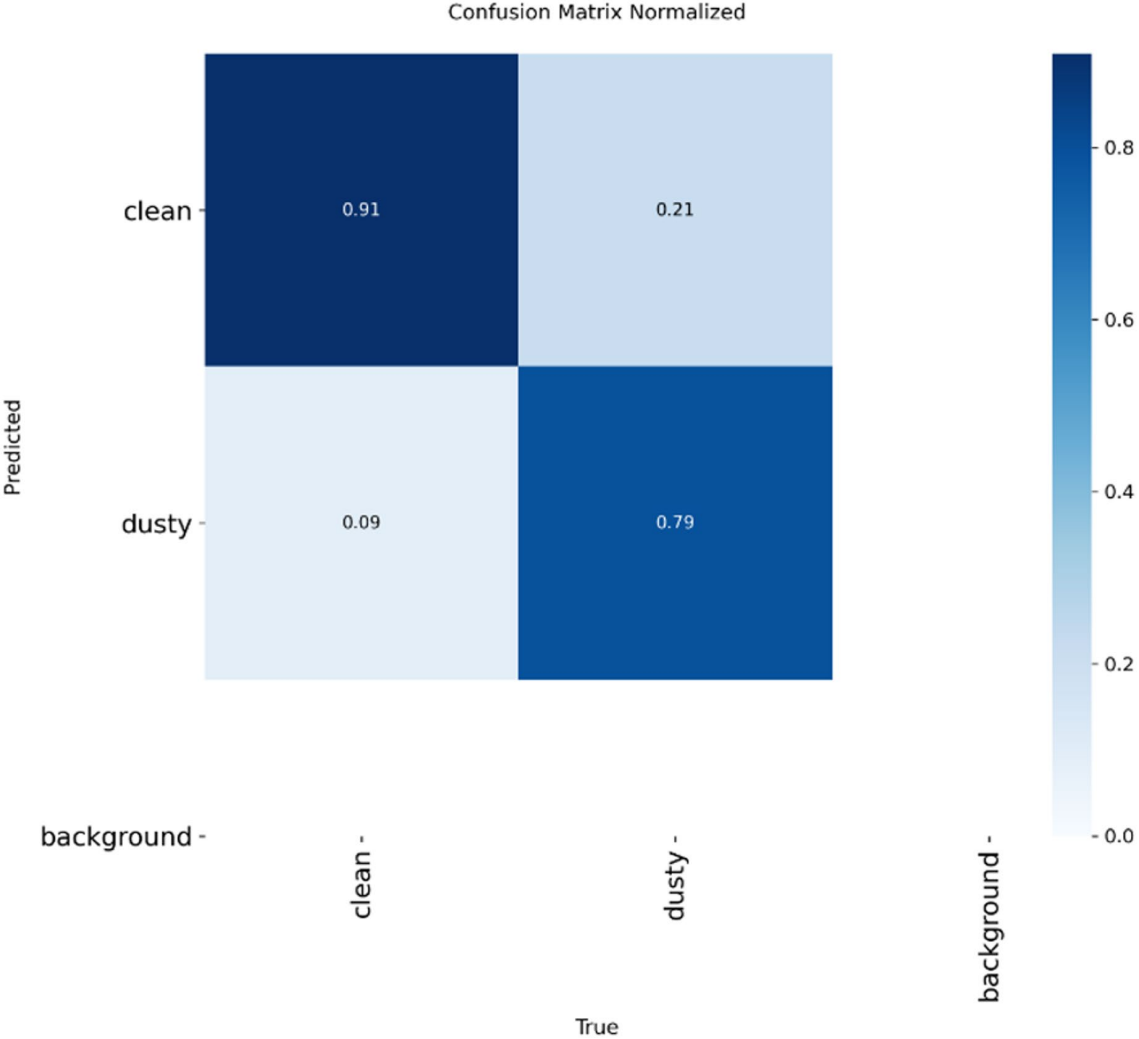
Normalized confusion matrix for YOLOv11x model.

The training process itself was monitored to ensure the models were learning effectively without significant overfitting. Figure 5 illustrates the progression of accuracy and loss for the YOLOv11x model over the training epochs, showing a stable convergence on both the training sets and validation sets. To help and gain a deeper insight into the classification accuracy of the top-performing model, the normalized confusion matrix was generated. The matrix in Fig. 6 visualizes the model’s predictions, distinguishing between correct classifications (True Positives and True Negatives) and errors (False Positives and False Negatives). For this project, a “False Negative”—classifying a dirty panel as clean—is the more critical error, as it would lead to a missed cleaning cycle and subsequent energy loss. The unique dataset strategy was instrumental to this success. By training the models exclusively on the most extreme cases—pristine panels and heavily soiled ones—we created a distinct and powerful learning signal. This binary clarity minimized ambiguity and enabled the models to achieve high confidence in their classifications. This approach perfectly mirrors the primary business logic of the system: to trigger a cleaning cycle when the panel condition is undeniably causing significant efficiency loss. However, this strategy also frames a key area for future investigation. The current models are experts at identifying the two ends of the spectrum but have not been explicitly trained on intermediate levels of dust accumulation. While the system works for triggering a much-needed cleaning, future iterations could involve a multi-class classification (e.g., ‘Clean’, ‘Lightly Dusted’, ‘Heavily Dusted’) or a regression model to predict the exact percentage of soiling. Such an enhancement would allow for an even more nuanced maintenance schedule, potentially recommending a less intensive “dry cleaning” for light dust, thereby further optimizing resource usage. Ultimately, the results from this section validate the core hypothesis of the concept. An accuracy of over 90% confirms that a camera, coupled with a well-trained AI model, can serve as a reliable sensor for solar panel soiling. This automates a critical data collection step that is traditionally manual and subjective, paving the way for the intelligent, performance-based cleaning system detailed in the following chapters. The successful visual detection is the first and most crucial trigger in a chain of logic designed to maximize energy output and minimize operational waste.

### Machine learning models performance results

**Fig. 7:**
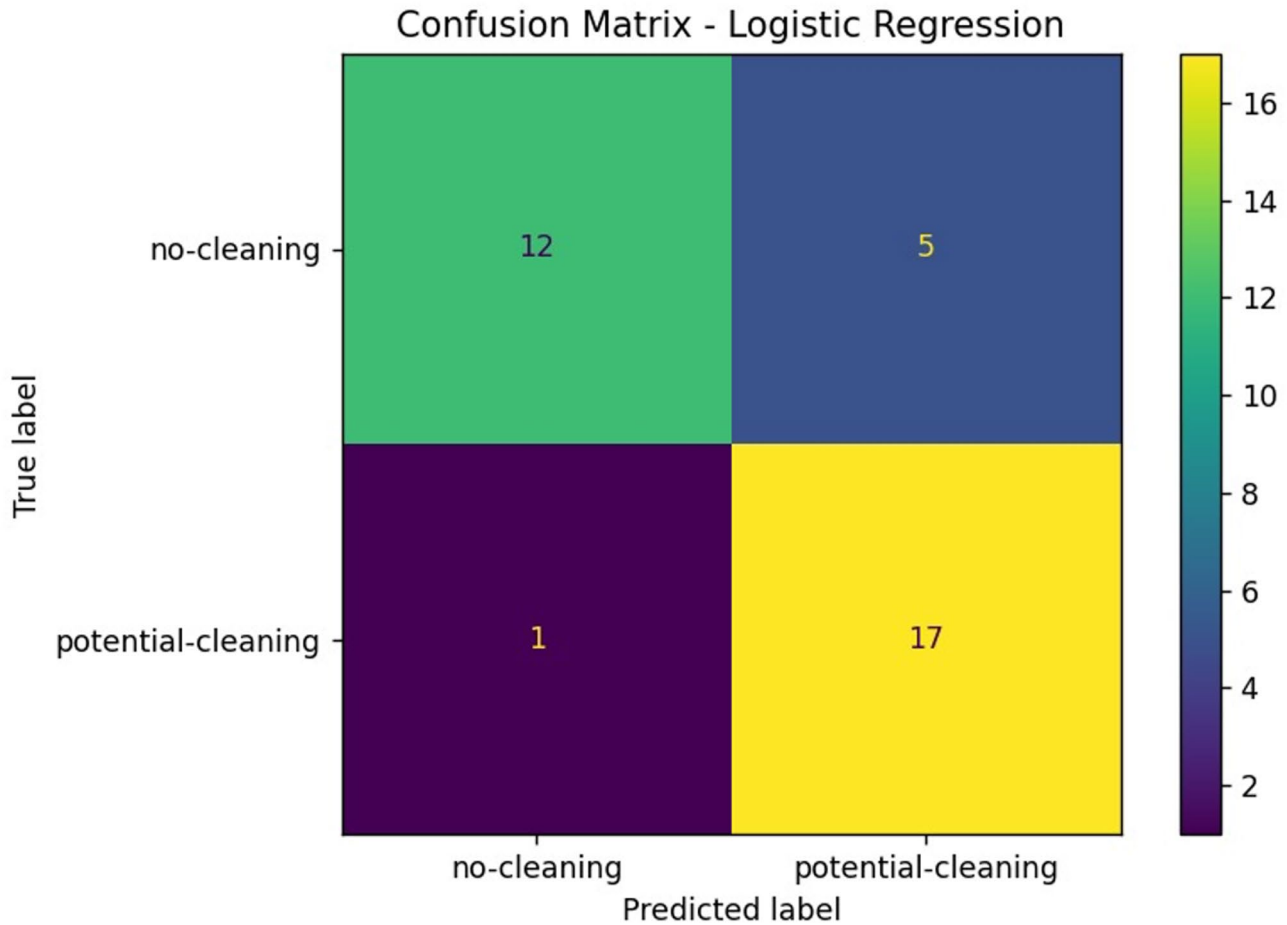
Confusion matrix—logistic regression.

While observing the confusion matrix for the LR model in Fig. 7, the performance analysis of the Logistic Regression model reveals several key observations. It accurately identified 17 out of 18 actual "potential-cleaning" panels, yielding a recall of approximately 0.94 for this class. This high recall is particularly valuable, as failing to identify a dirty panel could result in costly inefficiencies. However, the model also misclassified five "no-cleaning" panels as needing cleaning, which impacts the precision for the potential-cleaning class and raises operational costs due to potential over-maintenance. Despite having only one false negative—where a dirty panel was misclassified as clean—the implication of even one missed panel can be significant, depending on the deployment scale and subsequent energy loss. The model correctly identified 12 clean panels, resulting in a precision of approximately 0.92 for the "no-cleaning" class. This confusion matrix underscores the importance of high recall for the "potential-cleaning" class, even at the expense of increased false positives, a trade-off that is often acceptable in predictive maintenance systems because it minimizes the risk of missing cleanings. However, the presence of five misclassified clean panels suggests a possible bias toward predicting the “dirty” class, which may stem from class overlap or insufficient distinguishing features. Future improvements could involve integrating non-linear classifiers or additional features, such as weather conditions, to enhance the model’s discrimination capability. Overall, Logistic Regression achieved an accuracy of 0.83, with a precision of 0.92 and a recall of 0.71 for the "no-cleaning" class, leading to an F1-score of 0.80, whereas the "potential-cleaning" class exhibited a precision of 0.77 and a significantly improved recall of 0.94, resulting in an F1-score of 0.85. These metrics illustrate the typical trade-offs observed in imbalanced datasets; while the high precision for the "no-cleaning" class reflects that when the model predicts a panel does not need cleaning, it is usually correct, the lower recall indicates that several panels requiring cleaning are misclassified. The model’s strong recall in identifying "potential-cleaning" panels is critical, as overlooking such panels could lead to energy losses, reflecting the greater cost associated with false negatives compared to false positives in this context. The macro-averaged F1-score of 0.82 and the weighted average of 0.83 indicate reasonably balanced performance, albeit with some bias toward the "potential-cleaning" class. Nevertheless, the limitations of Logistic Regression as a linear classifier are acknowledged; it struggles to model complex, non-linear interactions among features. The decision boundary between clean and dirty solar panels is likely influenced by various environmental and physical factors, which makes Logistic Regression’s generalization capability potentially limited when faced with larger and more diverse datasets.

**Fig. 8:**
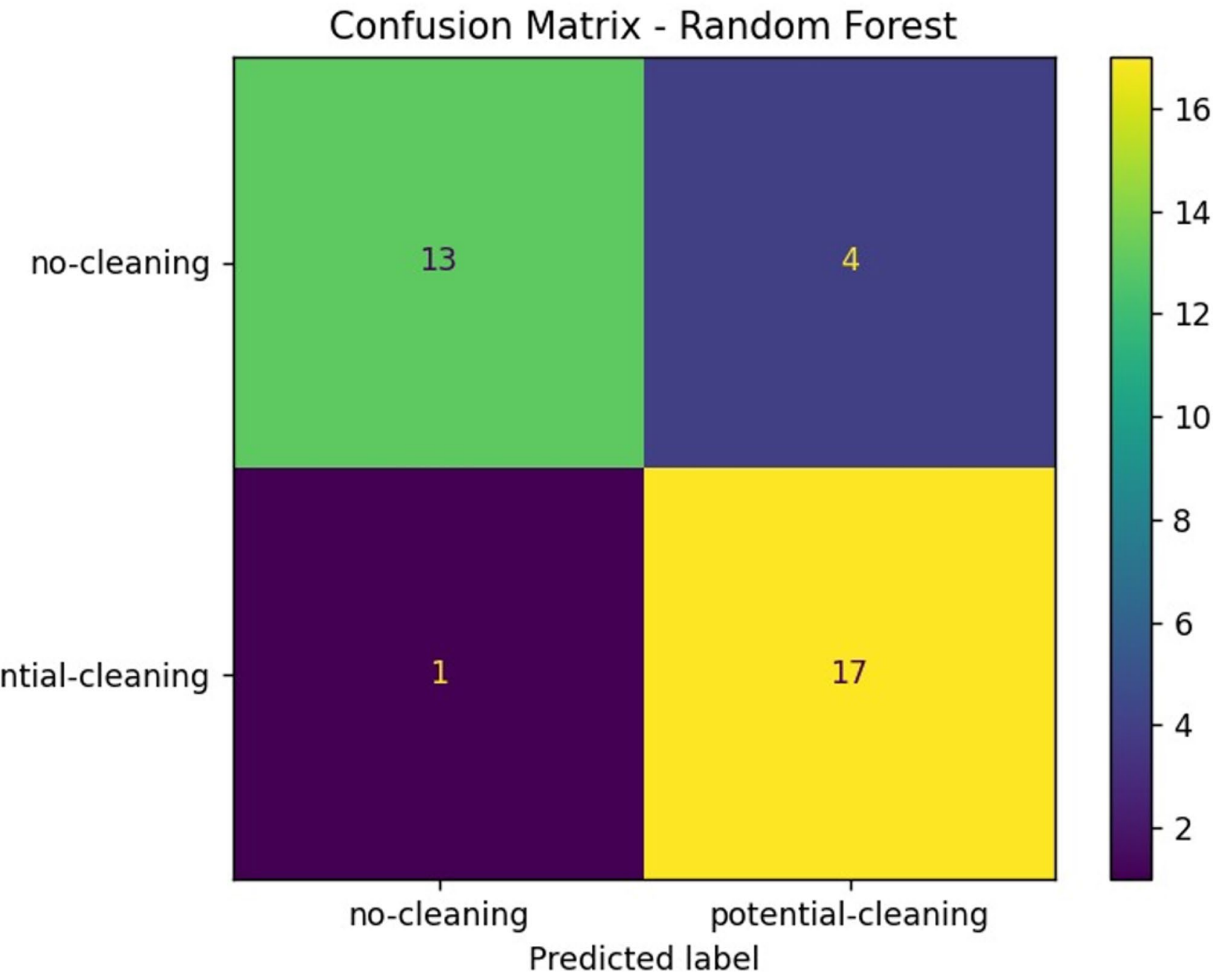
Confusion matrix—random forest.

In comparison, the Random Forest model matched Logistic Regression in accurately predicting 17 out of 18 "potential-cleaning" instances, resulting in a recall of approximately 0.94 for this critical class, while misclassifying only four "no-cleaning" panels, thereby reducing the number of false positives, see Fig. 8. This improvement in precision indicates a better balance between sensitivity and specificity. Like Logistic Regression, it only missed one dirty panel, reinforcing its reliability and reducing the risk of performance degradation due to undetected soiling. The Random Forest model correctly identified 13 out of 17 clean panels, contributing to a recall of approximately 0.76 and a precision of about 0.93 for the "no-cleaning" class, reflecting a slight enhancement over the Logistic Regression model. The enhanced performance of Random Forest, evident in its higher overall accuracy of 0.86, is attributed to its ability to model non-linear interactions and feature dependencies effectively; this capability is a marked advantage over the linear approach of Logistic Regression. The model attained a precision of 0.93 with a recall of 0.76 and an F1-score of 0.84 for the "no-cleaning" class, while its performance for the "potential-cleaning" class included a precision of 0.81, a recall of 0.94, and an F1-score of 0.87. This balanced performance suggests improved classification capabilities, allowing the Random Forest model not only to identify most dirty panels accurately but also to express confidence in these predictions, thus minimizing unnecessary maintenance actions. The strength of Random Forest lies in its ensemble approach, which aggregates the results of multiple decision trees to capture complex, non-linear patterns in the data, making it robust against noise and overfitting—an important feature given the small dataset size. The macro and weighted average F1-scores of 0.86 reflect consistent and reliable performance across all classes, reinforcing the model’s suitability for real-world deployment. However, the complexity of the Random Forest model may hinder interpretability compared to Logistic Regression, necessitating tools such as SHAP (SHapley Additive exPlanations) to explain decision-making processes, which introduces added complexity into the deployment pipeline. Additionally, with a limited number of training samples, there remains a risk of overfitting, emphasizing the importance of cross-validation and external testing on unseen data to confirm these results. In a comparative analysis, it is evident that Random Forest outperforms Logistic Regression, particularly in its ability to classify "potential-cleaning" panels accurately. In applications where the cost of missed cleanings is high—due to efficiency losses or contractual obligations—prioritizing high recall is critical. Both models achieved a strong recall for the "potential-cleaning" class (0.94), but Random Forest does this with improved precision, indicating fewer false alarms.

### Techno-economic inspection

**Fig. 9:**
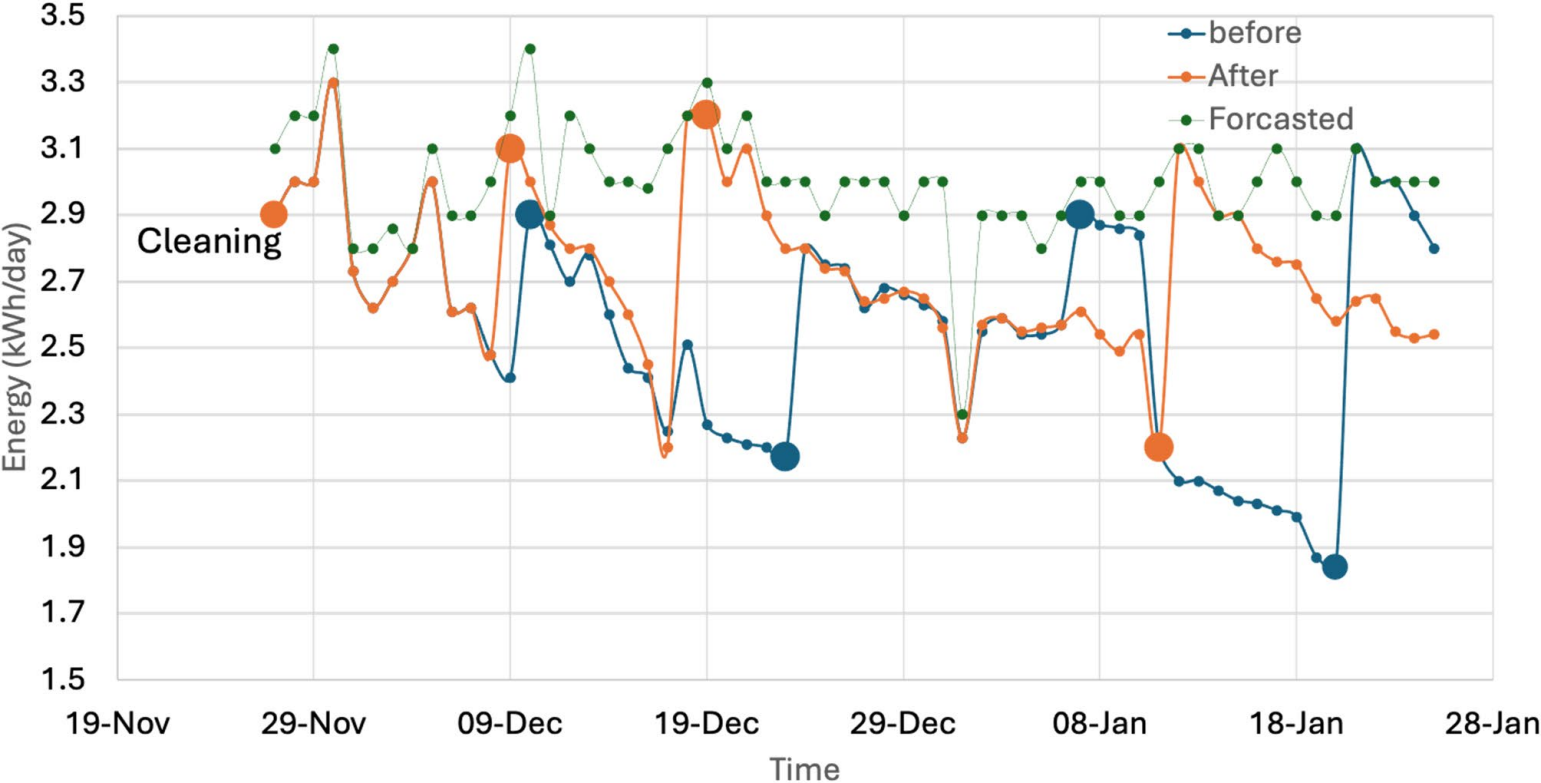
A sample of the energy yield for our proposed AI model against a bare system with a biweekly cleaning schedule.

As highlighted earlier in Section “Economic considerations”, the proposed AI-based model should be assessed from a techno-economic perspective to evaluate its performance against a bae system. Herein, we evaluate our system against a typical 5-kW system, as a bare system, for one entire year. Figure 9 displays a sample of two months of data for a bare system, a system connected to the cloud for dynamic cleaning decisions, and a forecasted dataset based on the irradiation level measured in the system location. The bare system applies a biweekly periodic cleaning model, as indicated in the graph in Fig. 9. Alternatively, the AI model applies a dynamic cleaning schedule based on the detected dust condition from the visualized dataset, as well as the raw data stream coming from the inverter. While in some cases, both the AI model and the bare system may agree on a specific cleaning date, the AI model showed its superiority in the sufficient cleaning procedure. For example, the AI system flags that a cleaning is needed on December 9^th^, while the periodic system needs to wait until the 10^th^ to make the scheduled cleaning. After only 9 days, the AI model flags another cleaning action, whereas the periodic model waits until December 24^th^. Alternatively, the periodic system operated a scheduled cleaning on January 7^th^, while the AI-based system doesn’t show a flag. A very interesting case appears on January 12^th^, because of a storm, the AI-dynamic system automatically flags an immediate cleaning action, while the bare system waits until the 21^st^. In other words, the system loses energy for around 10 days before it restores its default cleaned state.

Scaling up to annual evaluation, the proposed AI-model recorded ${\eta }_{cleaning}$ of 1.23, which reflects 23% enhanced energy productivity referring to the periodic cleaning case. Moving to the economic part, by applying Eq. (6), the system showed its capability to observe a saving of 2,023 USD. Herein, the benefits of showing a positive value for the saving parameter defined in Eq. (6) are not only a reflection of the economic feasibility of the model, but also prove the system efficiency to show a payback period in less than a year, as in Eq. (6), the entire system capital cost is included.

### GUI and user interaction

**Fig. 10:**
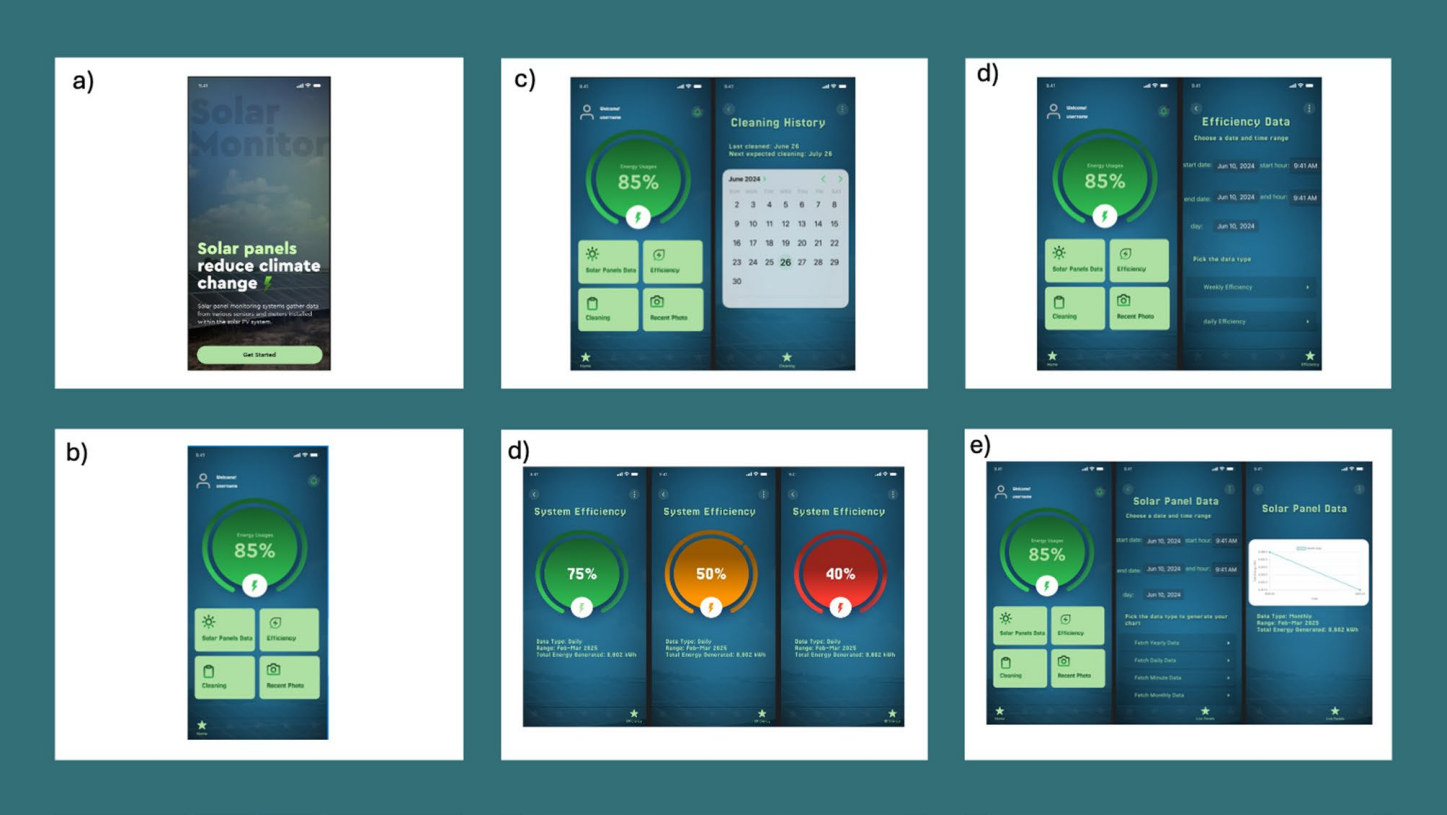
GUI for the developed mobile app showing (**a**) Home screen, (**b**) dashboard, (**c**) cleaning history, (**d**) system efficiency, (**e**) efficiency data, and (**f**) PV data.

Ensuring the system can be accessed conveniently as well as operated easily in real-world conditions for interaction, supervision, and decision support, we developed the WattsUp mobile application. Users can monitor the status of their solar panels, receive maintenance instructions, and analyze various data concerning energy and efficiency from their smartphones. The WattsUp application has been created in a way that it stands out and is instantly recognizable within the user’s mobile interface. The application itself gives a splash screen with the logo and other branding visuals, which creates a good impression for the users. This is more than just for looks; it provides an identity for the system, helping in user trust and consistent participation with the system. Users are welcomed by an onboarding interface that was shown in Fig. 10. This last screen explains the core purpose of the application, which is to monitor solar energy, and explains the overall importance of properly managing photovoltaic systems. The phrasing “Solar panels reduce climate change,” as well as other human-centered statements, draws a lot of attention. It allows users to connect instantly with the functionality of the system, which helps them understand the value of their interaction with the application.

After users authenticate, they are taken to the home dashboard, which is the main interface of the WattsUp application, as shown in Fig. 10b. This dashboard aims to give users an intuitive and visually appealing overview of the solar panel system’s status, prioritizing easy navigation and interaction, as well as real-time updates. Users receive a cumulatively computed value of their system’s operational performance and receive a circular progress indicator at the center of the screen, reminding them of the energy usage. Concise metrics like this support as an instantaneous feedback mechanism, helping users gauge whether their solar panels are working optimally. Four main navigation tiles are displayed: Solar Panels. Users can view their energy generation data and monitor their panel output for dust and equipment degradation issues over time. This feature gives access to current and historical, as well as dust and degradation-flagged, abnormal trending data. Efficiency Energy efficiency can be computed for any time with this feature, enabling users to view data and trends for any given time, including on a daily or weekly basis. It bolsters support for early detection of performance drops, aligning with the system’s core goal of predictive maintenance. Recent Photo: Displays the last recorded photo of the solar panel, allowing the user to assess visually if it is clean. This human verification step enhances transparency and trust in the automated model’s suggestions. These four core features were selected based on their usefulness for everyday monitoring, interaction with the model, and maintenance decision-making. By restricting the main dashboard to four features, cognitive overload is reduced, improving accessibility for both technical and non-technical users. The system design approach described earlier places the dashboard as an interface between the human factors and the intelligence embedded in the backend systems. It provides critical data to users exactly when they need it, supporting the system’s sustainability goals. With this design approach, even infrequent users can confidently engage in the passive and active management of their solar panel systems.

In this study, we utilized Python as the primary programming language due to its extensive ecosystem and versatility in handling tasks related to artificial intelligence, computer vision, and data analysis. Python’s readability and ease of use make it an ideal choice for rapid prototyping and development, enabling efficient experimentation with various machine learning algorithms. The framework employed for the deep learning models is TensorFlow, which was selected for its robustness and scalability, allowing the authors to leverage advanced neural network architectures such as YOLOv11. Additionally, essential libraries such as OpenCV were integrated for image processing tasks, providing powerful tools for handling image data and augmenting the visual dataset. The choice of this technology stack was driven by the need for efficient processing of large image datasets, the capability to deploy sophisticated machine learning models, and the requirement for a scalable solution that can adapt to future enhancements like edge AI integration. By organizing the GUI into these focused screens, the application not only streamlines user interaction but also ensures that users can easily access crucial information, effectively supporting the overarching goal of enhancing solar panel maintenance and promoting proactive energy management. This structured approach to GUI design ultimately contributes to a more engaged user experience and fosters better decision-making in solar energy management. The proposed system is still a prototype, but these plans allow for more effective deployment beyond the current prototype setup. For large-scale deployment on solar farms, system architecture can be augmented using two methods: fixed-position camera arrays and drone-based aerial imaging. Arrays of fixed cameras placed at specific intervals throughout a solar farm can provide uninterrupted visual coverage to monitor solar panels and their surfaces in real time. As an alternative, autonomous drones outfitted with high-resolution cameras can periodically scan wide regions, capturing oblique images of solar panels to assess the level of dust accumulation over expansive fields. As data collection continues over extensive periods of time, it becomes possible to improve the models in place using increasingly sophisticated methods such as deep learning, particularly LSTM networks that model complex temporal patterns for energy output. Moreover, an additional future improvement involves the incorporation of a method recommendation for cleaning, providing actionable advice based on the environmental conditions and state of the panels. Considering the amount of dust, drop in energy production, and weather forecast, the system could recommend either wet or dry cleaning, which would optimize efficiency while conserving resources, especially water. This approach allows for the best cleaning strategies to be applied while reducing operational costs and environmental harm.

The proposed system is currently demonstrated on a 5-kW prototype in Egypt; however, a more thorough discussion of its scalability and potential applications in utility-scale solar farms would significantly strengthen the paper. For instance, the model’s performance should be evaluated in larger solar installations, where the sheer volume of panels necessitates efficient monitoring and maintenance solutions. The integration of the proposed model with Supervisory Control and Data Acquisition (SCADA) systems could facilitate automated maintenance scheduling, ensuring timely cleaning interventions based on real-time data analytics. This integration would not only enhance operational efficiency but also streamline the overall management of solar farm operations. Furthermore, practical challenges must be addressed to realize this scalability. Camera calibration is critical for ensuring accurate dust detection and would require robust mechanisms to maintain consistent performance across varying environmental conditions. The implementation of drone-based inspections could offer a practical solution for large-scale monitoring; however, this approach would bring additional considerations regarding the costs and logistics of deploying hundreds of drones or camera nodes across extensive solar fields. Discussing these practical challenges and potential solutions would highlight the research’s significance beyond the prototype stage and illuminate paths for future research and real-world applications. Addressing these issues will be crucial for realizing the comprehensive integration of AI-driven solutions into the infrastructure of large-scale solar energy systems, ultimately enhancing their sustainability and operational effectiveness.

Despite the promising results and contributions of this study, several limitations warrant consideration. Firstly, the reliance on a specific dataset of 17,000 labeled images, while comprehensive, may not encompass the full variability of dust accumulation scenarios encountered in diverse geographical and environmental conditions, potentially limiting the generalizability of the model. Additionally, the current system primarily focuses on binary classification of dust presence, which may overlook nuanced levels of dust accumulation that could further optimize cleaning strategies. Furthermore, while the use of a Raspberry Pi camera provides a cost-effective solution for image capture, its resolution and image quality may not match that of more advanced imaging technologies, potentially impacting detection accuracy. Looking ahead, the integration of edge AI presents an exciting opportunity to enhance the system’s capabilities by facilitating real-time processing and analysis of data directly on-site, thereby improving responsiveness and reducing latency. By incorporating edge AI, future work could also explore advanced techniques such as federated learning, allowing for continuous model improvement through decentralized data collection from multiple solar installations while preserving user privacy. This approach not only addresses the limitations of the current study but also positions the system for scalability and adaptability in various operational contexts, ultimately driving further advancements in solar energy management.

## Conclusion

This study successfully developed an AI-driven solution for the early detection of dust accumulation on solar energy modules, addressing significant gaps in the literature and enhancing photovoltaic (PV) maintenance strategies. Key contributions include the utilization of a comprehensive visual dataset of 17,000 labeled images (10,300 dusty and 6,700 clean panels) and real-time energy production data from a 5-kW photovoltaic system, providing a robust foundation for model training and evaluation. The model evaluations revealed that the Random Forest classifier achieved an accuracy of 86%, with a precision of 93% and a recall of 76% for the "no-cleaning" class. In contrast, the YOLOv11x model demonstrated a remarkable accuracy of 90.7% in detecting dusty solar panels, showcasing its effectiveness for real-time applications. The dynamic cleaning model achieved a cleaning efficiency of 1.23, resulting in a 23% increase in energy production compared to traditional cleaning methods, translating to an estimated annual savings of $2,023. Notably, the system’s economic feasibility is underscored by a payback period of less than one year, highlighting the potential for intelligent solutions in solar energy management. Additionally, the WattsUp mobile application enhances user interaction by enabling monitoring of solar panel conditions and maintenance alerts, fostering a deeper connection between users and their PV systems.

Looking ahead, future work will focus on scaling the solution by incorporating UAV-based imaging for more extensive monitoring. We also plan to explore the integration of regression models to quantify dust accumulation levels more precisely. Expanding the dataset and implementing real-time model validation will further refine the system’s capabilities, alongside incorporating additional environmental factors, such as weather data, to enhance the optimization of solar panel maintenance strategies. This research lays a strong foundation for advancing the operational efficiency and sustainability of solar energy systems.

## Supplementary Information

Below is the link to the electronic supplementary material.

Supplementary Material 1

Supplementary Material 2

Supplementary Material 3

## Data Availability

Availability of data and material: The data supporting this study’s findings are available through: https://drive.google.com/drive/folders/1aDfDqh0XiCyq4Yqp7m9F6xhb8acvxh5l?usp=drive_link

## List of symbols

AUC-ROC: The area beneath the receiver operating characteristic curve
${Cap}_{node}$: The capital cost per node
CNN: Convolutional neural networks
${C}_{saving}$: The total annual savings after applying our proposed algorithm
${C}_{Tariff}$: The electricity tariff per kWh
${E}_{d,i}$: The daily energy yield in kWh after applying the dynamic model
${E}_{p,i}$: The daily energy yield in kWh for the bare periodic cleaning model
FN: False negatives
FP: False positives
GLCM: Gray-Level co-occurrence matrix
KNN: K-Nearest Neighbors’
LBP: Local binary pattern
LSTM: Long-short term memory
ML: Machine learning
MSE: Mean square error
${N}_{d}$: The number of cleaning processes through a year after applying the proposed model
${N}_{node}$: The number of hardware prototype nodes
${N}_{p}$: The total number of cleaning processes across the entire year
${OC}_{cleaning}$: The operational cost required for a single cleaning process, including the labor cost and other indirect costs, such as water consumption
${OC}_{cloud}$: Summaries the cloud storage and computing cost
PV: Photovoltaic
SVMs: Support vector machines
TN: True negative
TP: True positives
ViT: Vision transformer
${\eta }_{cleaning}$: An indicator to assess the dynamic cleaning model performance
