# Supplementary material for: Early detection of dust accumulation on solar energy modules using computer vision and machine learning techniques
Source: Sci Rep. 2026 Feb 12;16:6151. doi: 10.1038/s41598-026-37020-0 (PMC12902068; doi:10.1038/s41598-026-37020-0)
Supplement: Supplementary file 1 — Supplementary Material 1 [file 41598_2026_37020_MOESM1_ESM.pdf]

# Early Detection of Dust Accumulation on Solar Energy Modules Using Computer Vision and Machine Learning Techniques

Sara Hesham[1], Mohamed Elgohary[1], Mariam Massoud[1], Nouran Adel[1], Omar Elmahy[1] and Sameh Abdellatif[1,*]

[1]The Electrical Engineering department and FabLab, at the Centre for Emerging Learning Technologies, CELT, British University in Egypt (BUE), 11387, Cairo, Egypt.

This paper has yet to be submitted or presented elsewhere previously.
*Corresponding author: Sameh O. Abdellatif (email: sameh.osama@bue.edu.eg).
Address: The British University in Egypt, El-Sherouk City – Misr-Ismalia Desert Road, Postal No. 11837 - PO Box 43.
email: Sameh.osama@bue.edu.eg
Tel: +20 1222194077

## Supplementary Material

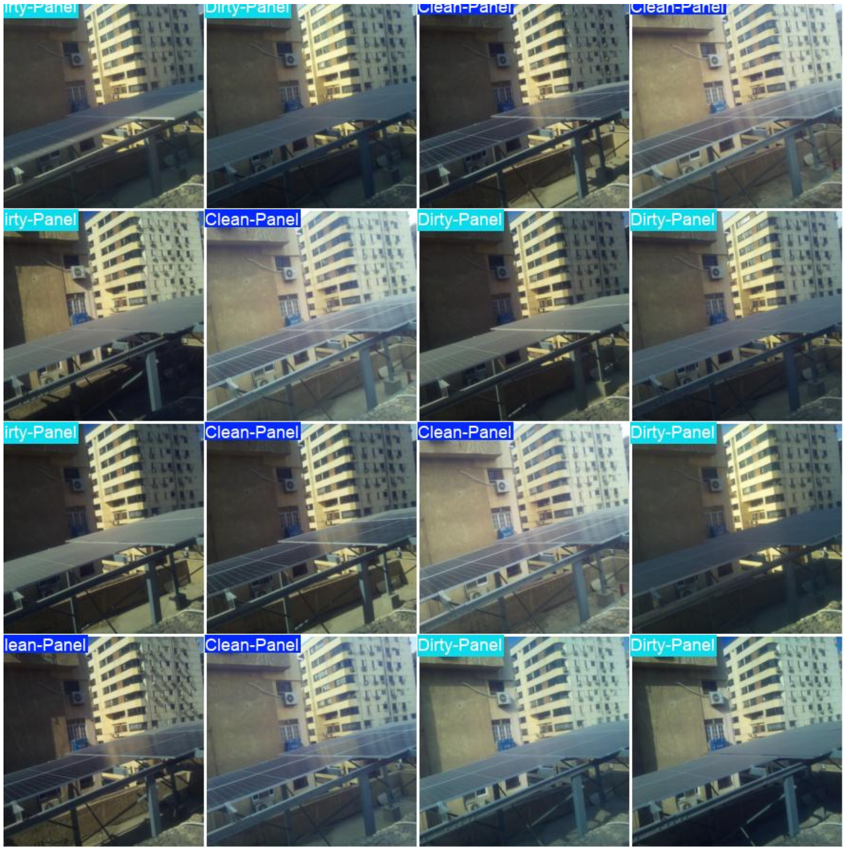


Figure S1: labeling process: Example 1

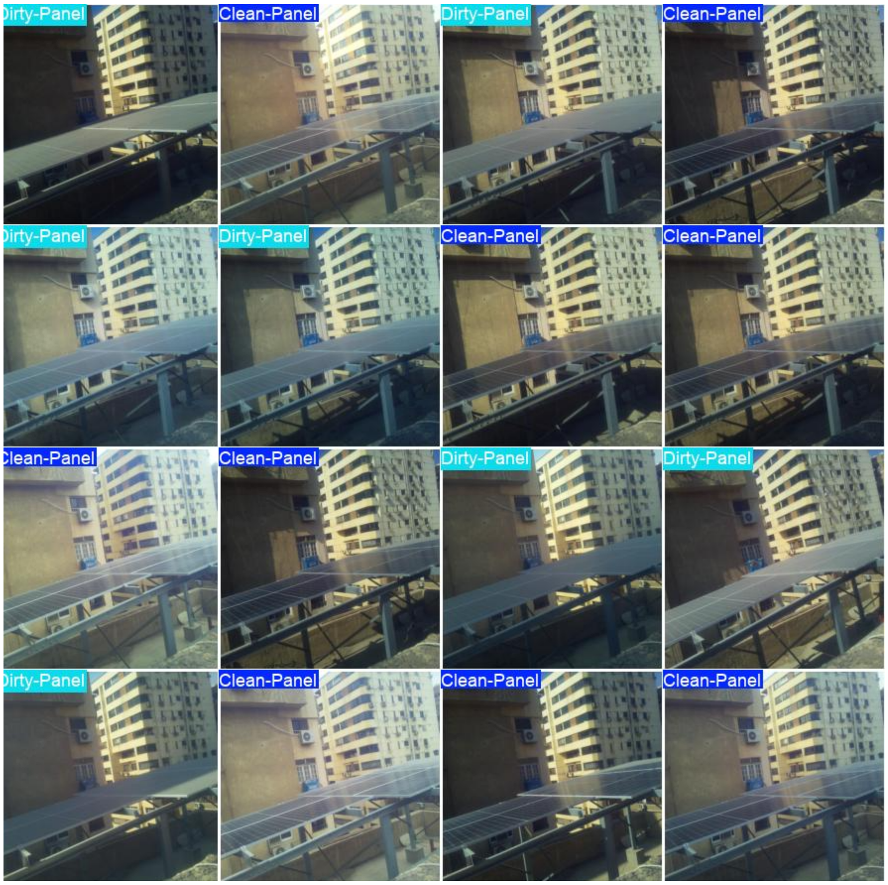


Figure S2: labeling process: Example 2

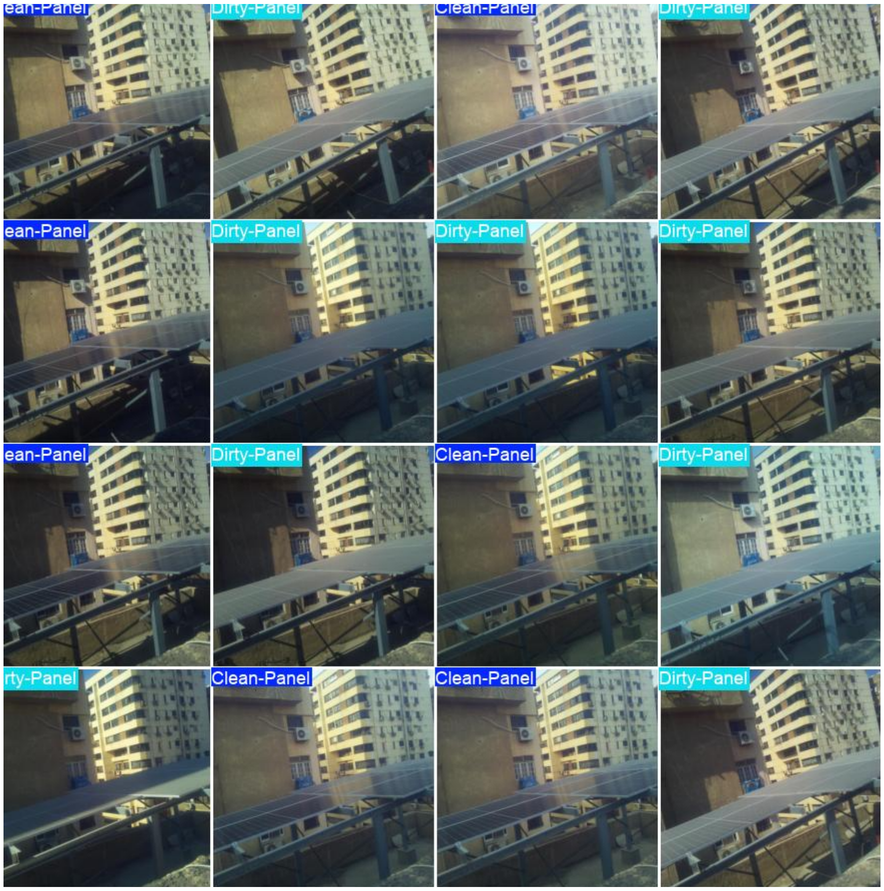


Figure S3: labeling process: Example 3
